# Analysis of factors affecting reversal of Hartmann’s procedure and post-reversal complications

**DOI:** 10.1038/s41598-020-73791-w

**Published:** 2020-10-08

**Authors:** Jae Hyun Kang, Byung Mo Kang, Sang Nam Yoon, Jeong Yeon Kim, Jun Ho Park, Bo Young Oh, Jong Wan Kim

**Affiliations:** 1 Department of Surgery, Dongtan Sacred Heart Hospital, Hallym University College of Medicine, 40, Sukwoo-Dong, Hwaseong-si, 445-170 Gyeonggi-do Republic of Korea; 2 Department of Surgery, Chuncheon Sacred Heart Hospital, Hallym University College of Medicine, Chuncheon-si, 200-950 Republic of Korea; 3 Department of Surgery, Kangnam Sacred Heart Hospital, Hallym University College of Medicine, 948-1, 1, Shingil-ro, Yeongdeungpo-gu, Seoul, 150-950 Republic of Korea; 4 Department of Surgery, Kangdong Sacred Heart Hospital, Hallym University College of Medicine, 445 Gil-1-dong, Gangdong-gu, Seoul, 134-701 Republic of Korea; 5 Department of Surgery, Hallym Sacred Heart Hospital, Hallym University College of Medicine, Anyang-si, 445-907 Republic of Korea

**Keywords:** Diseases, Gastroenterology, Medical research

## Abstract

Although Hartmann’s procedure (HP) is commonly used as emergency treatment for colorectal disease, the reversal of HP (HR) is infrequently performed. The aims were to evaluate the rate of HR and determine the factors predictive of achieving HR. We retrospectively reviewed the medical records of patients who underwent HP between January 2007 and June 2019 at six Hallym University-affiliated hospitals. Multivariable analysis was performed to identify which factors were independently associated with HR. In the study period, 437 patients underwent HP, and 127 (29.0%) subsequently underwent HR. Of these, 46 (35.9%) patients experienced post-HR complications. In multivariable analysis, an interval between HP and HR of > 6 months was associated with the only lower rate of post-HR complications. Multivariate analysis showed that HR was less likely in patients aged > 70 years, those with American Society of Anesthesiologists (ASA) class III or IV, elective surgery, those experiencing more than two HP-related complications, and those with a malignancy (an indication for HP). One-third of the patients underwent HR. Age > 70 years, ASA class III or IV, elective surgery, more than two HP-related complications, and malignancy were associated with a non-HR rate.

## Introduction

In 1921, Henry Albert Hartmann initially reported a surgical procedure for removing distal sigmoid colon cancer^1^. Hartmann’s procedure (HP) involves resection of the rectosigmoid colon and formation of the rectal stump and end colostomy.

This technique was initially used as emergency surgery for colorectal cancer complicated with perforation and obstruction^2,3^. It is often performed in patients with complicated diverticulitis, sigmoid volvulus, and traumatic colon injuries ^2,4–8^. Although primary anastomosis is attempted whenever possible, HP is still necessary in some patients with hemodynamic instability, panperitonitis, or extensive colorectal diseases that may introduce technical difficulties in single-stage procedures.

Although a recent Cochrane review reported no significant difference in quality of life after permanent colostomy compared with primary anastomosis, that review focused on patients with malignancies^9^. Other studies have suggested that HP causes considerable physical and psychological distress associated with the permanent colostomy^10,11^.

Reversal of HP (HR) involves closure of the end colostomy, mobilization of the proximal stump, and restoration of bowel continuity by stapling or a hand-sewn technique. HR is not performed in all patients after HP, such as those with dense adhesions or a short rectal stump^12^. Moreover, HR is associated with a significant morbidity rate of 22.9–68.4%^13–18^ and a mortality rate of 0–4.7%^4,14,17–22^. Several scoring systems have been proposed to predict whether HR is a good option for patients with acute diverticulitis^16,23,24^. Previous studies have reported predictive factors for achieving HR, including younger age, male sex, low American Society of Anesthesiologists (ASA) classification, and benign pathology^2,4,12–15,18–20^. However, there are no established guidelines for deciding whether or not to restore bowel continuity after HP. This decision is generally based on the surgeon’s discretion and the patient’s clinical condition.

This retrospective multicenter study was designed to evaluate the rate of HR and to identify which factors were predictive of HR over a 10-year period.

## Patients and methods

This retrospective study evaluated patients who underwent HP with or without HR at six Hallym University-affiliated hospitals (Hangang Sacred Heart Hospital, Kangnam Sacred Heart Hospital, Chuncheon Sacred Heart Hospital, Kangdong Sacred Heart Hospital, Hallym Sacred Heart Hospital, and Dongtan Sacred Heart Hospital) between January 2009 and August 2019. We also evaluated patients who underwent HP for the next 6 months, through to February 2020, to confirm the optimal reversal timing of 6 months. Patients were divided into two groups according to whether or not they underwent restoration of intestinal continuity, as the HR and non-HR groups. The Institutional Review Board of Dongtan Sacred Heart Hospital approved this study (IRB no. 2019-12-010) and complies with the Helsinki Declaration. Due to the retrospective nature of the study, informed consent was waived by the Institutional Review Board of Dongtan Sacred Heart Hospital who approved the study.

We included all patients aged > 18 years who underwent HP at any of the six abovementioned hospitals. We also included patients who underwent HP at another hospital and were transferred to one of the six hospitals for HR if detailed medical records for the HP were available. We excluded patients with incomplete data, those aged < 18 years, those who underwent colostomy without sigmoid colon resection, and those who underwent loop or transverse colostomy. The decision to perform HP during laparotomy was based on the surgeon’s experience as well as the patient’s abdominal condition and clinical status. The decision to perform HR was based on the patient’s preference and the attending surgeon’s opinion and experience after evaluating the patient’s clinical condition.

We collected the medical records from the initial HP and, if applicable, from the HR for each patient, including information on patient characteristics and perioperative outcomes. The patient characteristics included age, sex, body mass index, ASA class, history of surgery/cancer, comorbidities, and the indications for HP. The indications for HP included malignancy, diverticulitis, trauma, volvulus, stercoral ulcer, and colonic ischemia. Comorbidities included hypertension (HTN), diabetes mellitus (DM) (type 1 or 2), cardiovascular diseases (acute myocardial infarction and atrial fibrillation), pulmonary diseases (chronic obstructive pulmonary disease, asthma, and tuberculosis), cerebrovascular diseases (epidural hemorrhage, subdural hemorrhage, and cerebral/cerebellar infarction), endocrine diseases (adrenal diseases), nephrotic diseases (chronic renal failure and nephrotic syndrome), and psychiatric disorders (dementia, Parkinson’s disease, schizophrenia, mood disorders, and intellectual disability).

Perioperative outcomes included the length of postoperative hospital stay, elective /emergency surgery, postoperative complications, hospital readmission, reoperation, and mortality. Elective surgery was defined as a procedure that was performed on the scheduled date in patients with severe adhesion/inflammation, a huge/bulky tumor, or invasion of an adjacent organ or tissue by the tumor. Emergency surgery was defined as procedures performed in patients with unexpected or unstable clinical conditions, including hypovolemic shock or septic shock due to trauma or pan-peritonitis. Postoperative complications were defined as any condition that required an additional procedure or resulted in prolonged hospitalization, and included superficial/deep surgical site infection (SSI)^25^, organ/space SSI^25^, postoperative bleeding, ileus, an issue at the colostomy site (parastomal/incisional hernia), and respiratory (atelectasis, asthma, and chronic obstructive pulmonary disease), cardiac (acute myocardial infarction, congestive heart failure, and atrial fibrillation), nephrotic (acute kidney injury and acute renal failure), and psychotic (delirium and seizure) events. Complications were classified using the Clavien–Dindo classification^26^. Mortality was defined as death within 30 days after HP.

The primary endpoint of the study was to analyze the predictive factors of the reversal procedure. The secondary endpoint was to assess risk factor of the complication after HR.

### Statistical analysis

All statistical analyses were performed using SPSS version 24.0 (SPSS Inc., Chicago, IL, USA). Categorical variables were analyzed using the χ^2^ test or Fisher’s exact test and are presented as numbers and percentages of patients. Continuous variables were compared using the Mann–Whitney *U* test and are presented as means ± standard deviation. Multivariable logistic regression was performed to identify independent predictors for (1) post-HR complications and (2) undergoing HR. The confounding factors evaluated in the multivariate analyses included variables that were previously reported to be associated with HR and its complications. A *P*-value of < 0.05 was considered statistically significant.

## Results

A total of 437 patients underwent HP at one of six Hallym University-affiliated hospitals over the 10-year study period, and 127 (29.0%) subsequently underwent HR.

Table 1 lists the characteristics of the HR and non-HR groups. The mean age was 67.7 years, and patients in the non-HR group were significantly older than those in the HR group (70.2 vs. 61.6 years, *P* < 0.001). There were no differences between the two groups in terms of sex or body mass index. The proportion of patients with ASA class III or IV was much higher in the non-HR group than the HR group (55.2% vs. 43.9%, *P* = 0.034). At the time of surgery, 273 (62.6%) patients had at least one comorbidity and 27 (6.2%) had more than two comorbidities. The most common comorbidity was HTN (45.5%), followed by DM (19.0%) and cerebrovascular disease (10.8%), all of which were significantly more frequent in the non-HR group than in the HR group (50.3% vs. 33.9%, *P* = 0.002; 21.9% vs. 11.8%, *P* = 0.014; and 13.2% vs. 4.7%, *P* = 0.009, respectively).

**Table 1:** Patient characteristics between non-reversal and reversal groups after Hartmann’s procedure.

	All (n = 437)	Non HR (n = 310)	HR (n = 127)	*P*
Age (years)	67.7 (13.8)	70.2 (12.7)	61.6 (14.3)	< 0.001
Male	220 (50.3)	157 (50.7)	63 (49.6)	0.844
BMI (kg/m^2^)	22.8 (3.5)	22.6 (3.6)	23.4 (3.1)	0.058
**ASA**				0.034
I/II	208 (48.0)	139 (44.8)	69 (56.1)	
III/IV	225(52.0)	171 (55.2)	54 (43.9)	
Comorbidities	273 (62.6)	197 (63.8)	76 (59.8)	0.443
HTN	199 (45.5)	156 (50.3)	43 (33.9)	0.002
DM	83 (19.0)	68 (21.9)	15 (11.8)	0.014
Digestive disease	9 (2.1)	8 (2.6)	1 (0.8)	0.458
Pulmonolary disease	20 (4.6)	17 (5.5)	3 (2.4)	0.209
Cerebrovascular disease	47 (10.8)	41 (13.2)	6 (4.7)	0.009
Cardiologic disease	27 (6.2)	21 (6.8)	6 (4.7)	0.419
Nephrotic disease	9 (2.1)	8 (2.6)	1 (0.8)	0.458
Endocrinologic disease	29 (6.6)	22 (7.1)	7 (5.5)	0.546
Psychiatric disease	4 (0.9)	3 (1.0)	1 (0.8)	1.00
Others^a^	12 (2.7)	12 (3.9)	0 (0.0)	0.022
Three or more	27 (6.2)	22 (7.1)	5 (3.9)	0.210
Previous op history	127 (29.1)	39 (30.7)	88 (28.4)	0.627
Previous cancer history	53 (12.1)	45 (14.5)	8 (6.3)	0.017
**Diagnosis**				0.631
Malignancy	227 (51.9)	184 (59.4)	43 (33.9)	
Diverticulitis	66 (15.1)	35 (11.3)	31 (24.4)	
Trauma	38 (8.7)	15 (4.8)	23 (18.1)	
Fistula	7 (1.6)	6 (1.9)	1 (0.8)	
Severe adhesion	3 (0.7)	6 (1.9)	1 (0.8)	
Ischemia	62 (14.2)	41 (13.2)	21 (16.5)	
Volvulus	7 (1.6)	6 (1.9)	1 (0.8)	
Stercoral	21 (4.8)	17 (5.5)	4 (3.1)	
Others^b^	6 (1.4)	4 (1.3)	2 (1.6)	
**Tumor stage**				0.003
I	8 (3.8)	3 (1.6)	5 (11.6)	
II	68 (30.0)	54 (29.3)	14 (32.6)	
III	97 (42.7)	77 (41.8)	20 (46.5)	
IV	54 (23.8)	50 (27.2)	4 (9.3)	

Data are presented as the number of patients (%) or mean (standard deviation) unless otherwise stated.

*HR* Hartmann’s reversal *BMI* body mass index, *ASA* American Society of Anesthesiologists, *HTN* hypertension, *DM* diabetes mellitus, op operation.

^a^Others means benign prostate hyperplasia, rheumatoid arthritis.

^b^Others means fecal incontinence, rectal prolapse, ileus, adhesion band.

The outcomes after HP are shown in Table 2. The mean hospital stay was 22.8 days (range 1–149 days) and was not significantly different between the two groups (22.6 in the non-HR group vs. 23.4 days in the HR group, *P* = 0.685). In all patients, emergency HP was more frequent than elective HP (59.5% vs 40.5%). Elective HP was more frequent in the non-HR group than in the HR group (45.2% vs 29.1%, P = 0.002). The rate of HP-related complications was significantly greater in the non-HR group than in the HR group (47.1% vs. 33.1%, *P* = 0.007). In terms of specific complications, pulmonary complications were more frequent in the non-HR group vs. HR group (13.9% vs. 5.5%, *P* = 0.013), whereas psychotic complications were more frequent in the HR group vs. non-HR group (7.9% vs. 3.9%, *P* = 0.082). In terms of the Clavien–Dindo classification, the HR group experienced more severe complications (Grade III–V) than the non-HR group (P < 0.001). The proportion of patients with more than two HP-related complications was similar between the two groups (13.9% vs. 8.7%, *P* = 0.133). Forty-six (10.5%) patients died within 30 days after the initial HP because of septic shock, pneumonia/adult respiratory distress syndrome, or acute renal failure.

**Table 2:** Perioperative outcome between non-reversal and reversal groups after Hartmann’s procedure.

	All (n = 437)	Non-HR (n = 310)	HR (n = 127)	*P*
Duration of POD (days)	22.8 (20.0)	22.6 (19.7)	23.4 (20.8)	0.685
**Operation**				
Elective	177 (40.5)	140 (45.2)	37 (29.1)	0.002
Emergent	260 (59.5)	170 (54.8)	90 (70.9)	
Complications	188 (43.0)	146 (47.1)	42 (33.1)	0.007
Superficial/deep SSI	38 (8.7)	28 (9.0)	10 (7.9)	0.696
Organ/space SSI	31 (7.1)	24 (7.7)	7 (5.5)	0.410
Postoperative bleeding	4 (0.9)	2 (0.6)	2 (1.6)	0.583
Ileus	12 (2.7)	9 (2.9)	3 (2.4)	1.00
Stomy problem	5 (1.1)	5(1.6)	0 (0.0)	0.328
Pulmonary	50 (11.4)	43 (13.9)	7 (5.5)	0.013
Cardiology	19 (4.3)	14 (4.5)	5 (3.9)	0.788
Nephrology	14 (3.2)	10 (3.2)	4 (3.1)	1.000
Psychotics	22 (5.0)	12 (3.9)	10 (7.9)	0.082
Others^a^	60 (13.7)	52 (16.8)	8 (6.3)	0.004
**Clavien–Dindo classification**				< 0.001
Grade I	64 (34.0)	39 (26.7)	25 (58.1)	
Grade II	24 (12.7)	18 (12.3)	6 (13.9)	
Grade III	45 (23.9)	34 (23.3)	11 (25.6)	
Grade IV	9 (4.8)	8 (5.4)	1 (2.3)	
Grade V	46 (24.5)	46 (31.5)	0 (0.0)	
Complications ≥ 2	54 (12.4)	43 (13.9)	11 (8.7)	0.133
Mortality in 30 days	46 (10.5)	46 (14.8)	0 (0.0)	< 0.001

Data are presented as the number of patients (%) or mean (standard deviation) unless otherwise stated.

*HR* Hartmann’s reversal*, n* Numbers, *POD* postoperative days, *SSI* surgical site infection.

^a^Others means deep vein thrombosis, voiding difficulty, septic shock.

Table 3 shows the outcomes after HR. Out of 437 patients, 127 (29.0%) underwent HR. After excluding the 46 patients who died within 30 days after HP, the HR rate was 32.5%. The mean interval from HP to HR was 10.2 months (range 1–118 months). Two patients underwent HR almost 10 years after HP (118 and 112 months, respectively). After excluding these two patients, the mean interval from HP to HR decreased to 7.8 months (range 1–55 months).

**Table 3:** Postoperative outcome for Hartmann’s reversal.

	HR (n = 127)
Duration of POD (days)	16.5 (15.1)
Time to reversal (months)	10.2 (20.8)
Complications	46 (35.9)
Superficial/deep SSI	12 (9.4)
Organ/space SSI	5 (3.9)
Postoperative bleeding	1 (0.8)
Ileus	17 (13.3)
Incisional hernia at stomy site	11 (8.6)
Pulmonary	3 (2.3)
Psychotics	4 (3.1)
Others^a^	2 (1.6)
**Clavien–Dindo classification**	
Grade I	9 (19.5)
Grade II	17 (37.0)
Grade III	19 (41.3)
Grade IV	1 (2.1)
Complications ≥ 2	8 (6.3)
Readmission	11 (8.6)
Reoperation	15 (11.8)
Mortality in 30 days	1 (0.7)

Data are presented as the number of patients (%) or median (standard deviation) unless otherwise stated.

*HR* Hartmann’s reversal, *n* numbers, POD postoperative days, SSI surgical site infection.

^a^Others means deep vein thrombosis, voiding difficulty.

The post-HR hospital stay was 16.5 days (range 5–79 days). Post-HR complications occurred in 46 (35.9%) patients, and 8 (6.3%) patients experienced more than one complication. The most common complication was ileus (13.3%), followed by superficial/deep SSI (9.4%), incisional hernia at the colostomy site (8.6%), and organ/space SSI (3.9%). Eleven (7.8%) patients were re-admitted due to ileus (10 patients, 7.8%) or intra-abdominal abscess (1 patient, 0.8%), and 15 (11.8%) patients underwent reoperation due to an incisional hernia (10 patients, 7.8%), wound infection (1 patient, 0.8%), organ/space SSI (3 patients, 2.4%), or ileus (1 patient, 0.8%). One (0.8%) patient died within 30 days after HR because of a small bowel perforation related to panperitonitis. In terms of the Clavien–Dindo classification, Grade III complications were the most common (41.3%), followed by Grade II (37.0%), Grade I (19.5%), and Grade IV (2.1%) complications.

Univariate (P = 0.044) and multivariate (P = 0.027) analyses revealed that an interval of > 6 months between the initial HP and HR was associated with the only lower rate of post-HR complications (Table 4).

**Table 4:** Univariate and multivariate analysis of factors for complications after Hartmann’s reversal.

Variable	Univariate analysis		Multivariate analysis	
	OR (95% CI)	*P*	OR (95% CI)	*P*
Age > 70 years	0.670 (0.310–1.447)	0.307	0.513 (0.217–1.214)	0.129
Male	1.776 (0.854–3.695)	0.123	1.585 (0.735–3.419)	0.240
ASA III/IV	0.898 (0.428–1.884)	0.775	1.016 (0.459–2.250)	0.969
Comorbidities > 2	1.778 (0.109–29.111)	1.000	1.616 (0.089–29.301)	0.747
**Cause of HP**				
Malignancy	0.706 (0.322–1.551)	0.385	1.315 (0.448–3.861)	0.618
Time to reversal ≥ 6 mon	0.469 (0.223–0.985)	0.044	0.319 (0.115–0.881)	0.027

*OR* odds ratio, *CI* confidence interval, *ASA* American Society of Anesthesiologists, *HP* Hartmann’s procedure, *mon* months.

In univariate analysis, age > 70 years (P = 0.002), ASA class III/IV (P = 0.034), elective HP (P = 0.002), and malignancy (P < 0.001) were associated with a lower rate of HR (Table 5). In multivariate analysis, age > 70 years (P = 0.001), ASA class III/IV (P = 0.013), elective HP (P = 0.032), malignancy (P < 0.001), and number of HP-related complications ≥ 2 (P = 0.032) were significantly associated with a lower rate of HR.

**Table 5:** Univariate and multivariate analysis of factors associated with Hartmann’s reversal.

Variable	Univariate analysis			Multivariate analysis	
	OR (95% CI)	*P*		OR (95% CI)	*P*
Age > 70 years	0.506 (0.331–0.773)	0.002		0.439 (0.268–0.720)	0.001
Male	0.959 (0.635–1.450)	0.844		0.909 (0.571–1.446)	0.686
ASA III/IV	0.636 (0.418–0.969)	0.034		0.538 (0.331–0.875)	0.013
Comorbidities > 2	0.535 (0.198–1.444)	0.210		0.481 (0.168–1.372)	0.171
Elective operation	0.499 (0.320–0.778)	0.002		0.560 (0.330–0.952)	0.032
**Cause of HP**					
Malignancy	0.351 (0.228–0.540)	< 0.001		0.303 (0.182–0.505)	< 0.001
Postop Cx ≥ 2	0.589 (0.293–1.182)	0.133		0.431 (0.199–0.930)	0.032

*OR* odds ratio, *CI* confidence interval, *ASA* American Society of Anesthesiologists, *HP* Hartmann’s procedure, *Postop* postoperative, *Cx* complication.

## Discussion

In the present study, the rate of HR was 29.0%, which is similar to the previously reported rates (25.9–69%)^2,4,13–17,19,20,23,27,28^. The low HR rate might be related to high rates of death from septic shock, HP-related complications, and advanced colorectal cancer during the follow-up period after HP. After excluding patients who died within 30 days after HP, the HR rate in our study increased to 32.9%.

The post-HR complication rate in the present study was 35.9%, similar to the rates in previous studies (22.9–68.4%)^13–18^. In previous studies, the most common post-HR complication was wound infection (16.6–40%)^14,15,21,27^, which is likely due to the need for colostomy manipulation during HR^28^. In the present study, although wound infection was the second-most common complication, the rate of wound infections (9.4%) was relatively lower than the rates reported in previous studies. This may be because we included severe cases that underwent reoperation and surgical procedures. Ileus was the most common post-HR complication, and is mainly caused by severe adhesions in the abdominal cavity following HP. Several studies have reported risk factors for post-HR complications, including old age, ASA class > 2, current smoking, the surgeon’s specialization, and a history of radiation therapy^12,17,21,28–30^. The multivariable analysis revealed that an interval between HP and HR of > 6 months was the only independent factor associated with decreased post-HR complications (odds ratio: 0.319, 95% confidence interval: 0.115–0.881; P = 0.027). A longer interval might provide patients with more time to recover from sepsis and for bowel edema and inflammation to subside^31^.

There has been some debate regarding the optimal timing of HR. Although some studies have suggested an interval between HP and HR of approximately 3–4 months^32,33^, others recommended an interval of approximately 6 months^30^. Salem et al. reported that the rate of creating a second stoma was lower in patients who underwent early HR (< 6 months after HP) than in patients who underwent late HR (> 12 months after HP) (5% vs. 12.5%)^22^. The present study showed that the complication rate was lower in the late HR group (> 6 months) than in the early HR group (≤ 6 months) (27.4% vs 44.6%, P = 0.044). A longer interval may allow the patient to complete adjuvant chemotherapy and provide an opportunity to detect pelvic cancer recurrence before performing HR.

Previous studies have analyzed the relationship between age and the rate of HR. Hodgson et al. reported that age < 70 years was the only significant factor associated with performing HR^19^. Royo-Aznar et al. reported that patients aged < 69 years were more likely to undergo HR^13^ and Hess et al. reported that patients aged > 76 years were less likely to receive HR^20^. Despite the differing cut-off ages, these studies consistently showed that age is a significant predictor for HR. In the present study, patients aged > 70 years were less likely to undergo HR (*P* = 0.001), similar to these previous studies.

Sex was another significant factor associated with undergoing HR^2,4,14,18^. Several studies have shown that HR is more frequently performed in males than in females. This may be because males have a greater preference for HR than do females^2^, even though the narrow pelvis in males is an unfavorable anatomical factor. However, most of those studies were based on the results of univariate analyses, whereas we did not detect a significant sex difference in the HR rate in either univariate (*P* = 0.844) or multivariate (*P* = 0.686) analyses.

Several studies have suggested that patients with a lower ASA class are more likely to undergo HR, based on univariate analyses^2,13–16,19^. Similarly, we found that patients with ASA class III/IV were less likely to undergo HR (P = 0.013). The Charlson comorbidity index has also been proposed as a predictive factor for HR^13,28^. Fleming et al. and Royo-Aznar et al. proposed cut-off Charlson comorbidity indices of < 2 and < 6, respectively, for predicting whether HR is performed^13,28^. However, these previous studies showed that patients with a lower Charlson comorbidity index are more likely to undergo HR.

It has been reported that HR is performed more frequently in patients with benign diseases^4,14,18^. On the other hand, several studies reported that advanced malignancy, including Dukes stage IV, was associated with receiving HR^2,13,15^. Our multivariate analysis revealed that patients with a malignancy are less likely to undergo HR (*P* < 0.001), although we did not evaluate the malignancies according to stage.

Elective surgery was a predictor of non-reversal of HP^15^. The proportion of elective HP cases ranged from 20.1 to 37.6% among patients who do not undergo HR and from 5.9 to 14.7% among those who did undergo HR^4,13–15^. Although the proportions of patients who underwent elective HP in the non-HR (45.2%) and HR (29.1%) groups in our study were slightly higher than those in previous studies, elective HP was associated with non-reversal of HP in our multivariate analysis (P = 0.032). In patients who underwent elective surgery, it is possible that the surgeons had sufficient time to perform preoperative evaluations and the surgery was performed with patients in more optimal conditions. Accordingly, if the anastomosis was originally rejected and stoma was considered during initial surgery, the stoma was unlikely to be temporary because the initial situation was unchanged. Emergency HP was identified as an independent predictor for HR^4,13–15^. The proportion of emergency HP cases ranged from 85.3 to 94.1% among patients who subsequently undergo HR and from 62.4 to 78.7% among those who do not undergo HR^4,13–15^, indicating that emergency HP often leads to HR.

Several recent studies have reported the use of laparoscopic surgery for HR. Laparoscopic surgery has been associated with quicker recovery of bowel function, shorter hospital stay, and fewer postoperative complications^34,35^. Although the results of two systemic reviews^27,36^ were comparable with those from previous studies^34,35^, randomized clinical trials are required to define the safety and feasibility of laparoscopic HR. The rate of conversion to open surgery ranged from 7 to 19.5%, and the main reason for conversion was dense adhesions resulting from the initial HP^34,35^. Moreover, Mazeh et al. reported that conversion to open surgery was significantly influenced by marking the rectal stump during the initial HP and the presence of adhesions during HR^35^.

There are several limitations to our study. This was an observational, retrospective study, which may introduce inherent selection bias. As the decision to undergo HR was based on the surgeons’ experience and preference across six hospitals, this may have resulted in considerable variability. However, despite the lack of standard guidelines, our data, collected from six hospitals, showed consistent results in terms of predictive factors for HR and its complications in the multivariate analysis. Second, although this study included a larger number of patients compared with previous studies^2,12,17,19,20,28^, the relatively small number of patients may limit our ability to draw definitive conclusions. Third, the reasons for not performing HR were not recorded in the medical charts for some patients, which may distort the results since they could not be evaluated here.

The present study provides important data on the clinical outcomes and predictive factors for HR. The decision to undergo HR is based on the patient’s age, ASA classification, elective surgery, number of HP-related complications, and the indications for HP. One-third of patients who received HP subsequently underwent HR, with a complication rate of approximately 30%. Caution should be exercised in patients who undergo HR within 6 months after the initial HP to decrease the post-HR complication rate. HR was less likely in older patients, patients with a higher ASA class, elective surgery, patients with more than two HP-related complications, and patients with a malignancy (indication for HP).

## Data Availability

Not applicable.
